# *Neisseria gonorrhoeae* and extended-spectrum cephalosporins in California: surveillance and molecular detection of mosaic *penA*

**DOI:** 10.1186/1471-2334-13-570

**Published:** 2013-12-04

**Authors:** Severin Gose, Duylinh Nguyen, Daniella Lowenberg, Michael Samuel, Heidi Bauer, Mark Pandori

**Affiliations:** 1 San Francisco Department of Public Health, 101 Grove St. Rm. 419, San Francisco, CA 94102, USA; 2 California Polytechnic State University at San Luis Obispo, 1 Grand Ave, San Luis Obispo, CA 93405, USA; 3 Sexually Transmitted Disease Control Branch, Division of Communicable Disease Control, Center for Infectious Diseases, California Department of Public Health, 850 Marina Bay Pkwy, Richmond, CA 94804, USA; 4 Currently at PharmGKB, Standford University, CA, Stanford University 1501 California Avenue, Palo Alto, CA 94304, USA

**Keywords:** *Neisseria gonorrhoeae*, Antimicrobial resistance, Extended-spectrum cephalosporin, Mosaic *penA* allele, Real-time polymerase chain reaction

## Abstract

**Background:**

The spread of *Neisseria gonorrhoeae* strains with mosaic *penA* alleles and reduced susceptibility to extended-spectrum cephalosporins is a major public health problem. While much work has been performed internationally, little is known about the genetics or molecular epidemiology of *N. gonorrhoeae* isolates with reduced susceptibility to extended-spectrum cephalosporins in the United States. The majority of *N. gonorrhoeae* infections are diagnosed without a live culture. Molecular tools capable of detecting markers of extended-spectrum cephalosporin resistance are needed.

**Methods:**

Urethral *N. gonorrhoeae* isolates were collected from 684 men at public health clinics in California in 2011. Minimum inhibitory concentrations (MICs) to ceftriaxone, cefixime, cefpodoxime and azithromycin were determined by Etest and categorized according to the U.S. Centers for Disease Control 2010 alert value breakpoints. 684 isolates were screened for mosaic *penA* alleles using real-time PCR (RTPCR) and 59 reactive isolates were subjected to DNA sequencing of their *penA* alleles and *Neisseria gonorrhoeae* multi-antigen sequence typing (NG-MAST). To increase the specificity of the screening RTPCR in detecting isolates with alert value extended-spectrum cephalosporin MICs, the primers were modified to selectively amplify the mosaic XXXIV *penA* allele.

**Results:**

Three mosaic *penA* alleles were detected including two previously described alleles (XXXIV, XXXVIII) and one novel allele (LA-A). Of the 29 isolates with an alert value extended-spectrum cephalosporin MIC, all possessed the mosaic XXXIV *penA* allele and 18 were sequence type 1407, an internationally successful strain associated with multi-drug resistance. The modified RTPCR detected the mosaic XXXIV *penA* allele in urethral isolates and urine specimens and displayed no amplification of the other *penA* alleles detected in this study.

**Conclusion:**

*N. gonorrhoeae* isolates with mosaic *penA* alleles and reduced susceptibility to extended-spectrum cephalosporins are currently circulating in California. Isolates with the same NG-MAST ST, *penA* allele and extended-spectrum cephalosporin MICs have caused treatment failures elsewhere. The RTPCR assay presented here may be useful for the detection of *N. gonorrheoae* isolates and clinical specimens with reduced extended-spectrum cephalosporin MICs in settings where antimicrobial susceptibility testing is unavailable. In an era of increasing antimicrobial resistance and decreasing culture capacity, molecular assays capable of detecting extended-spectrum cephalosporin of resistance are essential to public health.

## Background

*Neisseria gonorrhoeae* is the second most commonly reported sexually transmitted infection in the United States [1]. *N. gonorrhoeae* has demonstrated a remarkable ability to acquire resistance to all the first-line antimicrobials previously used including sulfanilamides, penicillins, tetracyclines, macrolides, and fluoroquinolones [2-7]. Currently, extended-spectrum cephalosporins are the only first-line antimicrobials recommended for the empirical treatment of uncomplicated gonorrhea in many countries. In the United States, the prevalence of isolates with reduced susceptibility to cefixime (CFM) has resulted in dual treatment with ceftriaxone (CRO) plus azithromycin (AZM) or doxycycline being the only Centers for Disease Control and Prevention (CDC) recommended treatment regimen [8]. *N. gonorrhoeae* strains with reduced susceptibility to extended-spectrum cephalosporins have been reported worldwide [9-19] and treatment failures with oral and injectable extended-spectrum cephalosporins have been reported in Europe, Africa, Asia, Australia and North America [20-29]. Recently, extensively-drug resistant *N. gonorrhoeae* strains have also been reported in Japan [17], France [23] and Spain [30] that displayed high-level resistance to CFM and CRO.

*N. gonorrhoeae* isolates with reduced susceptibility to extended-spectrum cephalosporins have been linked to altered penicillin-binding protein 2, encoded by the *penA* gene. These *penA* alleles were termed “mosaic” because their DNA sequence appears to have been formed through homologous recombination events with other *Neisseria* spp. that are naturally resistant to extended-spectrum cephalosporins [10]. In 2009, isolates with mosaic *penA* alleles (XXXIV, XXXVIII) were first described in the United States and were associated with reduced susceptibility to CFM in San Francisco isolates [18]. *Neisseria gonorrhoeae* multi-antigen sequence typing (NG-MAST) revealed that these isolates were sequence type (ST) 1407, which has since become a highly prevalent multi-drug resistant strain in Europe [31] and Japan [32].

In the US, surveillance of *N. gonorrhoeae* antimicrobial resistance is conducted by the CDC Gonococcal Isolate Surveillance Program (GISP), which collects isolates from men presenting at public health clinics in 28 counties including four in California. Although extensive antimicrobial susceptibility testing data is collected about the phenotypic characteristics of these isolates [33,34], little is known about the genetics or molecular epidemiology of isolates with reduced susceptibility to extended-spectrum cephalosporins in the US. To investigate the association between strains harboring mosaic *penA* alleles and reduced extended-spectrum cephalosporin susceptibilities in California, *N. gonorrhoeae* isolates collected from the four California GISP sites in 2011 were analyzed. Minimum inhibitory concentrations (MICs) to CRO, CFM, cefpodoxime (CPD) and AZM were determined by Etest and categorized according to the 2010 CDC alert value breakpoints [35]. Isolates were then screened for the presence of a mosaic *penA* allele using real-time PCR (RTPCR) [36] and reactive isolates were subjected to DNA sequencing of their *penA* alleles and NG-MAST.

Detecting *N. gonorrhoeae* strains with reduced susceptibility to cephalosporins is a challenge in the U.S. The vast majority of *N. gonorrhoeae* infections are diagnosed using nucleic acid amplification testing (NAAT) methods where no live organism is available. In an era of increasing antimicrobial resistance and decreasing laboratory culture capacity, molecular assays capable of detecting markers of resistance are essential tools for public health surveillance. Therefore, we developed a novel RTPCR capable of specifically detecting the mosaic XXXIV *penA* allele and other closely related mosaic *penA* alleles that have been associated with extended-spectrum cephalosporin treatment failures [23,24,37] and validated its use on urethral isolates and clinical urine specimens.

## Methods

### Human subjects

Isolates and clinical specimens used in this study were collected for public heath surveillance and de-identified prior to antimicrobial susceptibility testing, thus this work was considered public health practice and was exempt from human subjects regulations [38]. This category of research is not considered human subjects research and is not subject to Institutional Review Board oversight.

### Isolate and specimen collection

During 2011, urethral isolates (N = 684) were collected from men visiting public health clinics participating in GISP in Los Angeles, San Francisco, San Diego and Orange counties in California as described previously [39]. De-identified *N. gonorrhoeae* positive urine specimens matched to urethral isolates with the mosaic XXXIV *penA* allele (N = 3), *N. gonorrhoeae* negative urine specimens (N = 24) and *N. gonorrhoeae* negative pharyngeal swab specimens (N = 20) were obtained from the San Francisco Department of Public Health Laboratory in 2012. Urethral isolates and matched urine specimens were collected during the same clinic visit.

### Etest

Etest antimicrobial susceptibility testing was completed on 678 isolates for CRO, 351 isolates for CPD, 332 isolates for CFM and 682 isolates of AZM using chocolate agar as described previously [39]. The lower numbers of isolates tested by CFM and CPD resulted from a mid-year change to the testing protocol where CPD was replaced by CFM in the antimicrobial susceptibility testing panel.

### Sample preparation

Isolates were prepared for *penA* DNA sequencing, RTPCR and NG-MAST by extracting DNA from 200 μL of liquid culture using the QIAamp DNA Mini Kit (Qiagen, Hilden, Germany) on the automated QIAcube platform and were stored at -40°C until used. Clinical specimens were prepared for RTPCR by extracting DNA from 200 μL of the APTIMA specimen buffer using the QIAamp DNA Mini Kit on the automated Qiacube platform and were stored at -40°C until used.

### Quality control

For every Etest batch, two *N. gonorrhoeae* isolates with predetermined MICs were included as quality control strains. MICs for Etest control strains were determined through repeat testing by the SFDPH laboratory. The SF-2010-09-09 control strain was non-reactive by the screening RTPCR and had non-alert value MICs to all four antimicrobials (CRO = 0.016 μg/mL, CPD = 0.016 μg/mL, CFM = 0.016 μg/mL, AZM = 0.032 μg/mL). The SF-2009-11-07 control strain was reactive by the screening RTPCR, had the mosaic XXXIV *penA* allele and had alert value MICs to the three extended-spectrum cephalosporins, but not AZM (CRO = 0.125 μg/mL, CPD = 1.5 μg/mL, CFM = 0.19 μg/mL, AZM = 0.032 μg/mL). Etest batches passed quality control if the MICs for both control strains were within ± 1 log_2_ of the predetermined MIC. For every RTPCR batch, extracted DNA from the SF-2009-11-07 was used as a positive control and a negative water control was included.

### Detection of mosaic *penA* alleles

A TaqMan RTPCR was used to detect *penA* alleles with the mosaic structure as described previously [36]. Briefly, 5 μL of extracted DNA from each isolate was used as the template in a 20 uL reaction mix containing LightCycler FastStart DNA Master HybProbe master mix (Roche Diagnostics, Mannheim, Germany) and 1 uM, 250 nM and 5 mM of primers, probe and MgCl_2_, respectively. Primers and probe used are shown in Table 1. The following amplification conditions were used: 2 min at 50°C, 10 min at 95°C, and 40 cycles of 15 s at 95°C and 60 s at 60°C. The samples were analyzed on a Roche LightCycler 2.0 and were declared positive if their curves rose above 2.0 fluorescence before cycle 35. RTPCR crossing points, the cycle at which the sample’s fluorescence rises above background, were automatically calculated using the Roche LightCycler 2.0 software package and the absolute quantitation setting.

**Table 1:** **Oligonucleotides used for RTPCR and amplification and sequencing of *penA, por *** **and *tbpB***

**Primer**	**DNA sequence**
Ochiai RTPCR forward	5′-GTTGGATGCCCGTACTGGG-3′
Ochiai RTPCR reverse	5′-ACCGATTTTGTAAGGCAGGG-3′
Ochiai RTPCR probe^a^	5′-CGGCAAAGTGGATGCAACCGA-3′
Modified RTPCR forward	5′-TCAATACGCCTGCCTATGAG-3′
Modified RTPCR reverse	5′-GCACATCCAAAGTAGGATAAACG-3′
*por* forward	5′-CAAGAAGACCTCGGCAA-3′
*por* reverse	5′-CCGACAACCACTTGGT-3′
*tbpB* forward	5′-CGTTGTCGGCAGCGCGAAAAC-3′
*tbpB* reverse	5′-TTCATCGGTGCGCTCGCCTTG-3′
*penA* forward 1st half	5′-GCATCAGGATAATAATAACGAGAAG-3′
*penA* reverse 1st half	5′-TGTAAGGCAGGGTATTGAAT-3′
*penA* forward 2nd half	5′-GTTGGATGCCCGTACTGGG-3′
*penA* reverse 2nd half	5′-CAGCCAAAGGGGTTAACTTGCTGAAC-3′

^a^Covalently bonded to a 5-6-carboxyfluorescein-3-6-carboxytetramethylrhodamine fluorophore.

### *PenA* DNA sequencing

Isolates reactive by the screening RTPCR were subjected to *penA* DNA sequencing as described previously [18]. Briefly, 5 μL of extracted DNA from each isolate was used as the template in a 20 μL reaction mix using LightCycler FastStart DNA Master HybProbe master mix (Roche Diagnostics, Mannheim, Germany). The *penA* gene was sequenced in two pieces with one amplification reaction covering the first half of the gene and another amplification reaction covering the second half. The primers used are shown in Table 1. The samples were analyzed on a Roche LightCycler 2.0 and the amplification products were purified using the QiaQuick PCR Purification Kit (Qiagen, Hilden, Germany) on the automated Qiacube platform. Sequencing was completed by MCLAB Inc. (South San Francisco, CA) and Elim Biopharmaceuticals (Hayward, CA) using the same primers that were used for amplification.

### NG-MAST

The *por* and *tbpB* genes from isolates with alert value cephalosporin MICs were amplified and sequenced as described previously [40]. Briefly, 5 μL of extracted DNA from each isolate was used as the template in a 20 uL reaction mix containing LightCycler FastStart DNA Master HybProbe master mix (Roche Diagnostics, Mannheim, Germany). The samples were analyzed on a Roche LightCycler 2.0 and the amplification products were purified using the QiaQuick PCR Purification Kit (Qiagen, Hilden, Germany) on the automated Qiacube platform. Sequencing was completed by MCLAB (South San Francisco, CA) and Elim Biopharmaceuticals (Hayward, CA) using the same primers that were used for amplification. NG-MAST sequence types (STs) were obtained by uploading trimmed sequences to the NG-MAST database at http://www.ng-mast.net.

### Detection of mosaic XXXIV *penA* alelle

A modified TaqMan RTPCR was developed to specifically amplify the mosaic XXXIV *penA* allele. The mosaic XXXIV, CI [23], XXXIV with T534A amino acid alteration [24] and X [37]*penA* alleles share sequence identity in the region detected by the modified RTPCR (bp 836 – bp 1066). Amplification specificity was accomplished by designing the forward primer with internal mismatches and a terminal 3′ mismatch to prevent the amplification of mosaic *penA* alleles detected in this study that were not associated with alert value extended-spectrum cephalosporin MICs (XXXVIII, LA-A) [see multiple sequence alignment shown in Additional file 1]. The reaction conditions used were identical to the screening RTPCR. Primers and probe used are shown in Table 1. Urine specimens (N = 27) and pharyngeal swab specimens (N = 20) were tested using the APTIMA Combo 2 (Gen-Probe, San Diego, CA) to determine whether *N. gonorrhoeae* was present prior to testing by the modified RTPCR.

### Data analysis

Isolates with alert value MICs were classified according to the 2010 CDC GISP alert value breakpoints, which were 0.125, 0.25, 0.25, and 2.0 μg/mL for CRO, CPD, CFM and AZM respectively [35]. For this study, *penA* genotypes were defined by 100% identity at the nucleotide sequence level. DNA sequences were trimmed and aligned using MEGA 5.05 and the TeXshade package in LaTeX. All sequences presented in this study are publicly available through Genbank at the National Center for Biotechnology Information (http://www.ncbi.nlm.nih.gov/genbank). Genbank accession numbers for mosaic *penA* alleles XXXIV, XXXVIII, LA-A and wild-type (LM306) are [Genbank:GU723422], [Genbank:HQ204565], [Genbank:KC192769] and [Genbank:M32091], respectively.

## Results

### Phenotypic surveillance of 684 isolates collected in 2011

Overall, 15/678 isolates displayed alert value CRO MICs, 21/351 isolates displayed alert value CPD MICs and 2/682 isolates displayed alert value AZM MICs. No isolates with an alert value CFM MIC were observed and both isolates with alert value AZM MICs had extended-spectrum cephalosporin MICs below the alert value breakpoints.

### Genotypic surveillance of mosaic *penA* alleles in 684 isolates collected in 2011

Overall, 59/684 isolates were reactive by the screening RTPCR, indicating the presence of a mosaic *penA* allele. DNA sequencing revealed that 39 isolates possessed the mosaic XXXIV *penA* allele, four isolates possessed the mosaic XXXVIII *penA* allele and sixteen isolates possessed a novel mosaic *penA* allele that will be referred to as LA-A. Of isolates reactive by the screening RTPCR, 15/59 displayed an alert value CRO MIC and 21/59 displayed an alert value CPD MIC.

**Figure 1:**
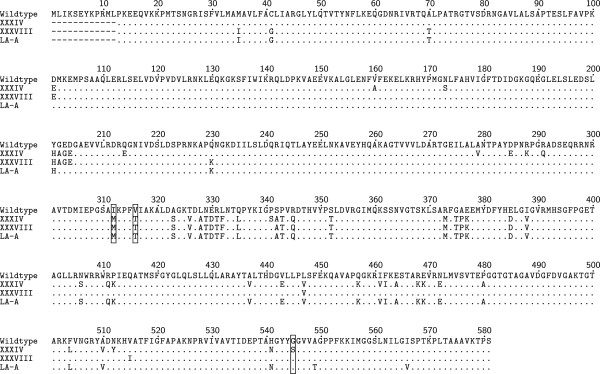
**Multiple alignment of translated amino acid sequences of mosaic *penA *alleles.** Comparison of the translated amino acid sequences for isolates with mosaic *penA* alleles detected in this study (XXXIV, XXXVIII and LA-A) to the wild-type sequence (LM306). Boxed residues represent amino acid changes associated with reduced susceptibility to oral extended-spectrum cephalosporins.

Figure 1 shows a full length multiple alignment of the translated amino acid sequences of mosaic *penA* alleles detected in this study. The mosaic XXXIV *penA* allele has all three amino acid changes (I312M, V316T, and G545S) that have previously been associated with reduced susceptibility to oral extended-spectrum cephalosporins [37,41]. The mosaic XXXVIII and LA-A *penA* alleles both have two of these three amino acid changes (I312M, V316T). None of the mosaic *penA* alleles found in this study have the recently described amino acid change (A501P) found in the mosaic CI *penA* allele from an extensively-drug resistant gonococcal strain that displayed high level resistance to both CFM and CRO [23,30].

### Association of the mosaic XXXIV *penA* allele with alert value extended-spectrum cephalosporin MICs

**Figure 2:**
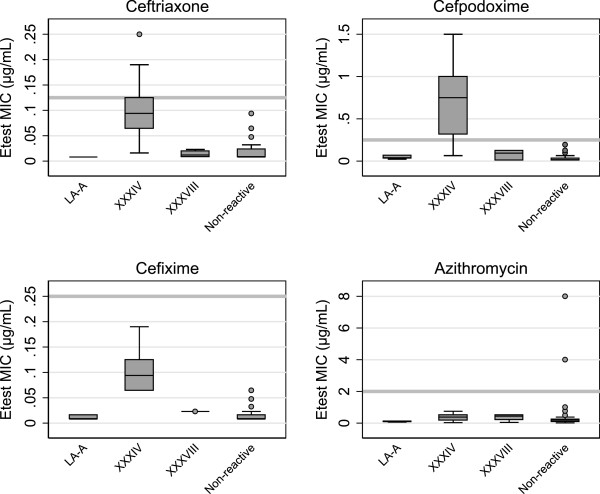
**Box plots of MIC distributions by *penA *genotype.** N = 678, 351, 332 and 682 for CRO, CPD, CFM and AZM respectively. Boxes indicate the 25th, 50th, and 75th percentiles. Whiskers indicate the lowest datum within 1.5 IQR of the lower quartile, and the highest datum within 1.5 IQR of the upper quartile. Outliers are shown as gray circles. Gray horizontal bars indicate the alert value breakpoint for each antimicrobial.

Figure 2 shows box plots of MIC distributions by *penA* genotype. The mosaic XXXIV *penA* allele was associated with elevated MICs for all three extended-spectrum cephalosporins, although for CRO and CFM, the majority of MICs remained below the alert value breakpoints. Of isolates with the mosaic XXXIV *penA* allele, 15/39 displayed an alert value CRO MIC and 21/24 displayed an alert value CPD MIC. All isolates with the mosaic XXXVIII, mosaic LA-A and non-reactive *penA* alleles had non-alert value extended-spectrum cephalosporin MICs. Both isolates that displayed alert value AZM MICs were non-reactive by the screening RTPCR and had non-alert value extended-spectrum cephalosporin MICs.

### NG-MAST of 59 isolates with mosaic *penA* alleles

Table 2 shows the *por* alleles, *tbpB* alleles and NG-MAST sequence types (STs) of isolates with a mosaic *penA* allele. Of the 39 isolates with the mosaic XXXIV *penA* allele, 24 were ST1407 (*tbpB* 110, *por* 908). In isolates with the mosaic XXXIV *penA* allele and STs other than ST1407, *tbpB* allele 110 was found in every isolate except one, which had the closely related *tbpB* allele 1431 (99.5% sequence identity with *tbpB* 110). Of the four isolates with the mosaic XXXVIII *penA* allele, all were ST1407. Of the 16 isolates with the mosaic LA-A *penA* allele, 10 were ST7268 (*tbpB* 18, *por* 4340). In isolates with the mosaic LA-A *penA* allele and STs other then 7268, *tbpB* allele 18 was found in three isolates, one isolate had *tbpB* allele 1429 (93.1% sequence identity with *tbpB* 18) and one isolate had *tbpB* allele 1430 (94.6% sequence identity with *tbpB* 18).

**Table 2:** **NG-MAST sequence types of isolates with a mosaic *penA *** **allele in California**

**Total no.**	**No. CRO alert**^**a**^	***penA *** **genotype**	***tbpB *** **allele**	***por allele***	**NG-MAST ST**
24	10	XXXIV	110	908	1407
1	0	XXXIV	110	1388	2212
2	0	XXXIV	110	1903	3149
1	1	XXXIV	110	2622	4275
1	0	XXXIV	110	2684	4378
1	0	XXXIV	110	3424	5643
4	0	XXXIV	110	2700	5895
1	1	XXXIV	110	3669	6200
2	2	XXXIV	110	4544	7560
1	0	XXXIV	110	4599	7647
1	1	XXXIV	1431^b^	1900	7566
4	0	XXXVIII	110	908	1407
10	0	LA-A	18	4340	7268
2	0	LA-A	18	4546	7564
1	0	LA-A	18	4545	7646
1	0	LA-A	81^c^	4340	7561
1	0	LA-A	1429^d^	4340	7562
1	0	LA-A	1430^e^	4547	7565

^a^CRO alert value MIC ≥ 0.125 μg/mL.

^b^*tbpB* allele 1431 is 2 bp different from *tbpB* allele 110.

^c^*tbpB* allele 81 is 27 bp different from *tbpB* allele 18.

^d^*tbpB* allele 1429 is 1 bp different from *tbpB* allele 18.

^e^*tbpB* allele 1430 is 21 bp different from *tbpB* allele 18.

### Modified RTPCR for the detection of the mosaic XXXIV *penA* allele

Table 3 shows results of the modified RTPCR performance in detecting the mosaic *penA* XXXIV allele in a validation panel of 71 *N. gonorrhoeae* isolates, 27 urine specimens and 20 pharyngeal swab specimens. The modified RTPCR was reactive in 39/39 urethral isolates containing the mosaic XXXIV *penA* allele and 3/3 urine specimens that were matched to urethral isolates possessing the mosaic XXXIV *penA* allele. No reactivity was seen in isolates with the mosaic XXXVIII, LA-A or *penA* alleles non-reactive by the screening RTPCR. All *N. gonorrhoeae* negative urine specimens and pharyngeal swab clinical specimens were also non-reactive. Based upon testing of the validation panel of 71 isolates, the sensitivity and specificity of the modified RTPCR in detecting alert value CRO MICs were 100% and 57% respectively.

**Table 3:** Isolates and clinical specimens tested with the modified RTPCR

**Specimen type**	**Total no.**	**No. CRO alert**^**a**^	**Source**	***penA *** **genotype**^**b**^	**NAAT result**^**c**^	**RTPCR result**^**d**^
Isolate	39	15	Urethral	XXXIV	+	+
	6	0	Urethral	XXXVIII	+	-
	6	0	Urethral	LA-A	+	-
	20	0	Urethral	Non-reactive	+	-
Clinical Specimen	3	ND	Urine	XXXIV	+	+
	24	ND	Urine	ND	-	-
	20	ND	Pharynx	ND	-	-

^a^CRO alert value MIC ≥ 0.125 μg/mL.

^b^ND = Not determined.

^c^Tested by APTIMA Combo 2.

^d^Tested by the modified RTPCR presented in this study.

## Discussion

*N. gonorrhoeae* has an extensive history of antimicrobial resistance and has demonstrated that it is capable of utilizing a variety of mechanisms to escape antimicrobial pressure. Extended-spectrum cephalosporins have been the CDC recommended treatment only since 2007, but it appears that their usefulness is already on the decline. In 2010, the CDC issued treatment guidelines recommending dual therapy with CFM plus AZM or doxycyline due to concerns about increasing antimicrobial resistance. In 2012, the CDC again updated its recommendations and abandoned oral extended-spectrum cephalosporins in favor of dual therapy with CRO plus AZM or doxycycline. The data here support this recommendation since no isolates were found with alert value MICs to both AZM and an extended-spectrum cephalosporin. Two isolates with alert value MICs to AZM were detected in San Francisco and San Diego, however, so the emergence of dual-resistant strains in California remains a possibility.

Three mosaic *penA* alleles were detected in this study and a novel one was identified. The mosaic XXXIV and XXXVIII *penA* alleles were both previously detected in San Francisco in 2009 [18], while the LA-A *penA* allele is novel. The mosaic XXXIV *penA* allele has all three amino acid changes previously associated with reduced susceptibility to oral extended-spectrum cephalosporins [37]. Homology modeling studies have shown that these amino acid changes result in conformational alterations of the β-lactam-binding pocket and affect the ability of extended-spectrum cephalosporins to bind, particularly those with large R groups [42]. More recently, it has been shown that these amino acid changes are epistatic and only result in higher MICs in the context of other mutational changes in *penA*[41]*.* Although the mosaic XXXVIII and LA-A *penA* alleles both have two of these amino acid changes, the distributions of extended-spectrum cephalosporin MICs for isolates with these *penA* alleles were similar to those with non-reactive *penA* alleles. These data support the hypothesis that the I312M and V316T amino acid changes act synergistically with the G545S amino acid change and that they are necessary, but not sufficient, to produce elevated oral extended-spectrum cephalosporin MICs.

The mosaic XXXIV *penA* allele detected in this study is concerning because it was found in isolates with extended-spectrum cephalosporin MICs that have been reported in isolates associated with treatment failures. Since first being published in 2009, the mosaic XXXIV *penA* allele has been found worldwide and is now associated with ST1407 strains and reduced susceptibility to extended-spectrum cephalosporins. A pharyngeal ST1407 isolate with the mosaic XXXIV *penA* allele recently caused a CRO treatment failure in Slovenia [29] and strains with closely related mosaic *penA* alleles have caused treatment failures in France (mosaic CI) [23], Austria (mosaic XXXIV with T534A amino acid alteration) [24] and Hong Kong (mosaic X) [37]. The mosaic XXXIV *penA* allele is only a single amino acid change (A501P) different from the mosaic CI *penA* allele described in extensively-drug resistant isolates from France and Spain that exhibited high-level resistance to both oral and injectable extended-spectrum cephalosporins [23,30]. This additional mutation might be capable of increasing CRO MICs from the elevated levels seen in this study (CRO MIC = 0.094-0.25 μg/mL) to levels at which CRO treatment failures have occurred even with much higher 1 g doses (CRO MIC = 1.0-2.0 μg/mL) [38]. The extensively-drug resistant isolates described in France and Spain were also ST1407, indicating a genetically similar background. It is possible, however, that determinants of resistance other than *penA* also affect the extended-spectrum cephalosporin MICs observed.

In 2009, the mosaic XXXIV and XXXVIII *penA* alleles were described only in ST1407 isolates and a very closely related isolate (ST1513, *tbpB* 110, *por* 971, 99.8% similar to *por* 908) in San Francisco and appeared to be spreading in a clonal fashion. The mosaic XXXVIII *penA* allele has remained clonal within ST1407 in California, while the mosaic XXXIV *penA* allele was found in 10 STs other than ST1407 in 2011. In isolates with the mosaic XXXIV *penA* allele and STs other than ST1407, the *tbpB* allele was the same as ST1407 (*tbpB* allele 110) or was a closely related *tbpB* allele (*tbpB* allele 1431, 99.5% similar to *tbpB* 110). ST1407 remains the dominant ST associated with reduced extended-spectrum cephalosporin susceptibilities in California, but strains with unrelated STs now carry the mosaic XXXIV *penA* allele. Genomic mapping of ST1407 and ST5895 isolates from San Francisco recently detailed the transfer of the mosaic XXXIV *penA* allele into a completely different genetic background [43]. In this study, four ST5895 isolates with the mosaic XXXIV *penA* allele were found in Los Angeles, Orange County and San Diego, indicating that this strain is likely circulating throughout California. Unfortunately, the horizontal spread of the mosaic XXXIV *penA* allele into new STs makes tracking the spread of extended-spectrum cephalosporin resistance with NG-MAST problematic.

Of note is the fact that the mosaic XXXIV *penA* allele was only found in isolates with *tbpB* allele 110 or a very closely related allele (*tbpB* 1431, 99.6% similar to *tbpB* allele 110). In the *N. gonorrhoeae* genome, *penA* is located ~50 kb downstream of *tbpB*. The linkage between these two loci likely explains the high level of clonality of *tbpB* alleles in isolates with the mosaic XXXIV *penA* allele. The linkage between *penA* and *tbpB* was also evident in isolates with the mosaic LA-A *penA* allele where 13/16 isolates had *tbpB* 18.

Detecting strains with reduced susceptibility to extended-spectrum cephalosporins is a challenge in the U.S. The vast majority of *N. gonorrhoeae* infections are diagnosed using nucleic acid-based methods where no live organism is present. Without a culture and antimicrobial susceptibility testing, molecular assays are currently the only option available for detecting isolates with reduced susceptibility to extended-spectrum cephalosporins. The modified RTPCR presented here is a simple and rapid tool capable of specifically detecting isolates or urine specimens that may have alert value extended-spectrum cephalosporin MICs. The assay may find utility in the surveillance and epidemiological investigation of strains with reduced susceptibility to extended-spectrum cephalosporins.

This assay, however, has several limitations. The mosaic *penA* alleles which the assay was designed to discriminate between were detected in California in 2011. It is possible that other mosaic *penA* alleles are circulating elsewhere that produce elevated extended-spectrum cephalosporin MICs, but do not share the region of sequence identity that the modified RTPCR detects. Also, the modified RTPCR should be used with caution on pharyngeal specimens because of the potential for cross-reactivity with *penA* alleles from other *Neisseria* spp. The assay performed well on *N. gonorrhoeae* positive urine specimens matched to isolates with the mosaic XXXIV *penA* allele, but only a small number of specimens were available for testing.

The main limitation of this study is the sampling strategy used to collect *N. gonorrhoeae* isolates. The samples were collected in parallel with the CDC GISP which collects isolates only from men. Additionally, the samples were collected from public health clinics whose populations may not be representative of the general population and are known to oversample MSM [44]. Since MSM populations have historically been associated with higher rates of antimicrobial resistance, it is possible that these data overestimate the prevalence of alert value isolates in the general population [34]. Another limitation was the use of RTPCR to screen isolates for the presence of a mosaic *penA* allele. The screening RTPCR detected a region of shared sequence identity found in many mosaic *penA* alleles, but it is possible that other mosaic alleles were present that are non-reactive with this assay.

## Conclusion

In conclusion, *N. gonorrhoeae* isolates with mosaic *penA* alleles and reduced susceptibility to extended-spectrum cephalosporins are currently circulating in California. Isolates with the same NG-MAST ST, *penA* allele and extended-spectrum cephalosporin MICs have caused verified treatment failures elsewhere. Enhanced surveillance efforts such as the use of the molecular assays to detect resistance determinants and the implementation of test of cure protocols will be necessary to ensure that empirical treatment remains effective.

## Abbreviations

AZM: Azithromycin; CDC: US Centers for Disease Control and Prevention; CFM: Cefixime; CPD: Cefpodoxime; CRO: Ceftriaxone; GISP: Gonococcal Isolate Surveillance Program; MIC: Minimum inhibitory concentration; NAAT: Nucleic acid amplification test; NG-MAST: *Neisseria gonorrhoeae* multi-antigen sequence typing; RTPCR: Real-time polymerase chain reaction; ST: Sequence type.

## Competing interests

The authors declare no competing interests.

## Authors’ contributions

SG completed the antimicrobial susceptibility testing, RTPCR screening, *penA* DNA sequencing, NG-MAST, developed and validated the modified RTPCR and prepared the manuscript. DN assisted with NG-MAST and validation of the modified RTPCR. DL assisted with NG-MAST. MS and HB consulted on the project and manuscript preparation. MP directed the project and consulted on the manuscript preparation and modified RTPCR design. All authors read and approved the final manuscript.

## Pre-publication history

The pre-publication history for this paper can be accessed here:

http://www.biomedcentral.com/1471-2334/13/570/prepub

## Supplementary Material

Additional file 1 **Multiple alignment of bp 791 – bp 1110 of the mosaic *penA *alleles detected in this study.** The locations of the primers and probe for both the screening and modified RTPCRs are shown. The modified forward primer specifically amplifies the mosaic XXXIV *penA* allele due to the internal mismatches and terminal 3′ mismatch with the other *penA* alleles detected in this study. Click here for file
