# Supplementary material for: Neisseria gonorrhoeae and extended-spectrum cephalosporins in California: surveillance and molecular detection of mosaic penA
Source: BMC Infect Dis. 2013 Dec 4;13:570. doi: 10.1186/1471-2334-13-570 (PMC4235022; doi:10.1186/1471-2334-13-570)
Supplement: Additional file 1 — Multiple alignment of bp 791 – bp 1110 of the mosaic penA alleles detected in this study. The locations of the primers and probe for both the screening and modified RTPCRs are shown. The modified forward primer specifically amplifies the mosaic XXXIV penA allele due to the internal mismatches and terminal 3′ mismatch with the other penA alleles detected in this study. [file 1471-2334-13-570-S1.pdf]

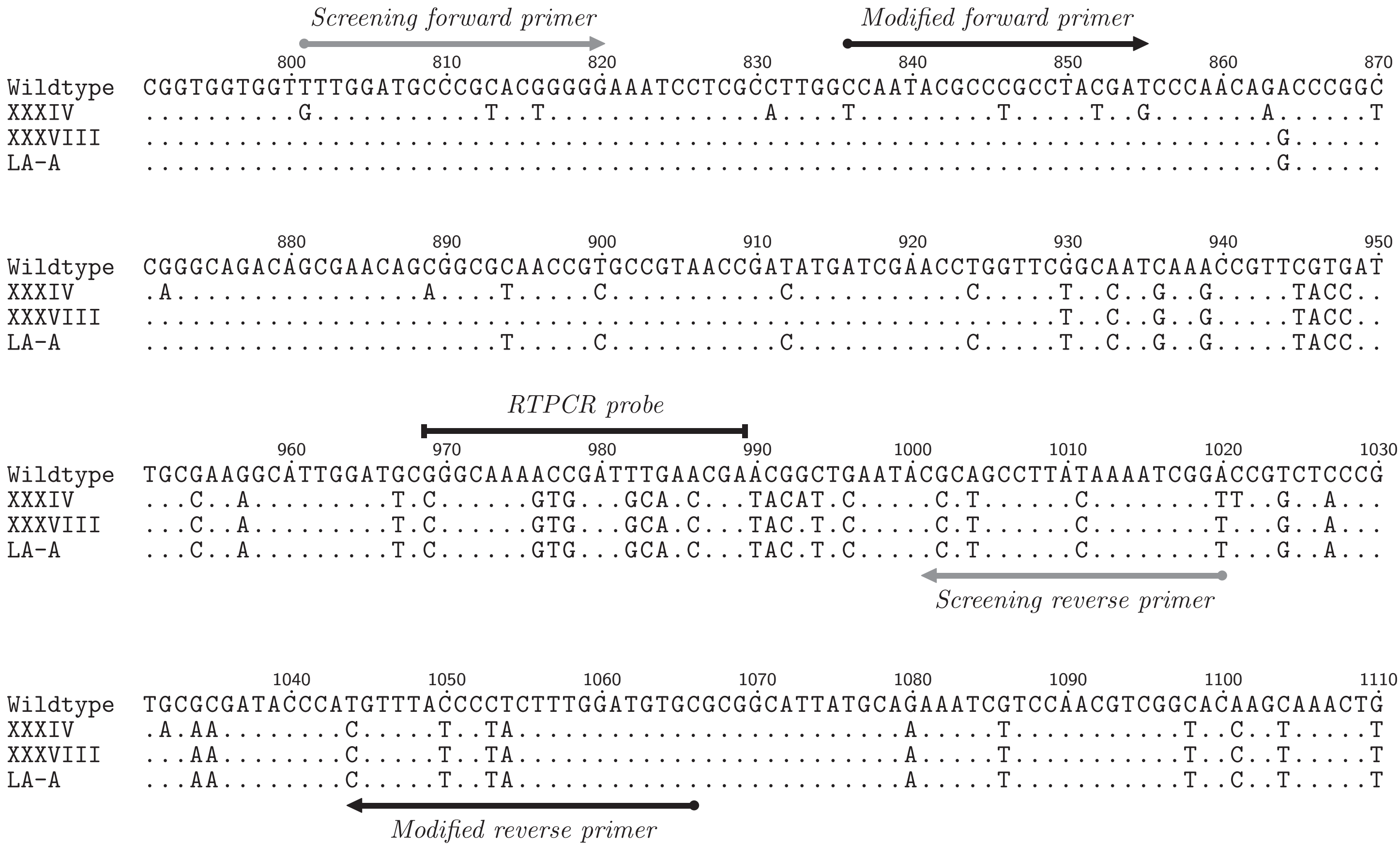
Screening forward primer
Modified forward primer
800 810 820 830 840 850 860 870
Wildtype CGGTGGTGGTTTTGGATGCCCGCACGGGGGAAATCCTCGCCTTGGCCAATACGCCCGCCTACGATCCCAACAGACCCGGC
XXXIV ..........G...........T..T..............A....T.........T.....T..G.......A......T
XXXVIII ....G......
LA-A ....G......
880 890 900 910 920 930 940 950
Wildtype CGGGCAGACAGCGAACAGCGGCGCAACCGTGCCGTAACCGATATGATCGAACCTGGTTCGGCAATCAAACCGTTCGTGAT
XXXIV .A................A....T.....C...........C...........C.....T..C..G..G.....TACC..
XXXVIII ....T..C..G..G.....TACC..
LA-A .......................T.....C...........C...........C.....T..C..G..G.....TACC..
RTPCR probe
960 970 980 990 1000 1010 1020 1030
Wildtype TGCGAAGGCATTGGATGCGGGCAAAACCGATTTGAACGAACGGCTGAATACGCAGCCTTATAAAATCGGACCGTCTCCCG
XXXIV ...C..A.........T.C......GTG...GCA.C...TACAT.C.....C.T......C........TT..G..A...
XXXVIII ...C..A.........T.C......GTG...GCA.C...TAC.T.C.....C.T......C........T...G..A...
LA-A ...C..A.........T.C......GTG...GCA.C...TAC.T.C.....C.T......C........T...G..A...
Screening reverse primer
1040 1050 1060 1070 1080 1090 1100 1110
Wildtype TGCGCGATACCCATGTTTACCCCTCTTTGGATGTGCGCGGCATTATGCAGAAATCGTCCAACGTCGGCACAAGCAAACTG
XXXIV .A.AA........C.....T..TA.........................A.....T...........T..C..T.....T
XXXVIII ...AA........C.....T..TA.........................A.....T...........T..C..T.....T
LA-A ...AA........C.....T..TA.........................A.....T...........T..C..T.....T
Modified reverse primer
